# Myelomonocytic Skewing In Vitro Discriminates Subgroups of Patients with Myelofibrosis with A Different Phenotype, A Different Mutational Profile and Different Prognosis

**DOI:** 10.3390/cancers12082291

**Published:** 2020-08-14

**Authors:** Klaus Geissler, Bettina Gisslinger, Eva Jäger, Roland Jäger, Ana-Iris Schiefer, Edith Bogner, Elisabeth Fuchs, Fiorella Schischlik, Donat Alpar, Ingrid Simonitsch-Klupp, Robert Kralovics, Heinz Gisslinger

**Affiliations:** 1 Medical School, Sigmund Freud University, 1020 Vienna, Austria; 2 Department of Internal Medicine V with Hematology, Oncology and Palliative Care, Hospital Hietzing, 1130 Vienna, Austria; 3 Division of Hematology and Hemostaseology, Department of Internal Medicine I, Medical University of Vienna, 1090 Vienna, Austria; bettina.gisslinger@meduniwien.ac.at (B.G.); heinz.gisslinger@meduniwien.ac.at (H.G.); 4 Department of Laboratory Medicine, Medical University of Vienna, 1090 Vienna, Austria; eva.jaeger@akhwien.at (E.J.); rkralovics@cemm.oeaw.ac.at (R.K.); 5 CeMM Research Center for Molecular Medicine of the Austrian Academy of Sciences, 1090 Vienna, Austria; roland.jaeger@meduniwien.ac.at (R.J.); edith_bogner@hotmail.com (E.B.); elisabeth.fuchs@edumail.at (E.F.); fiorella.schischlik@nih.gov (F.S.);; 6 Clinical Institute of Pathology, Medical University of Vienna, 1090 Vienna, Austria; ana-iris.schiefer@meduniwien.ac.at (A.-I.S.); ingrid.simonitsch-klupp@meduniwien.ac.at (I.S.-K.)

**Keywords:** myelofibrosis, skewing, progenitor cells, in vitro culture, hematopoiesis, prognosis

## Abstract

Normal hematopoietic function is maintained by a well-controlled balance of myelomonocytic, megaerythroid and lymphoid progenitor cell populations which may be skewed during pathologic conditions. Using semisolid in vitro cultures supporting the growth of myelomonocytic (CFU-GM) and erythroid (BFU-E) colonies, we investigated skewed differentiation towards the myelomonocytic over erythroid commitment in 81 patients with myelofibrosis (MF). MF patients had significantly increased numbers of circulating CFU-GM and BFU-E. Myelomonocytic skewing as indicated by a CFU-GM/BFU-E ratio ≥ 1 was found in 26/81 (32%) MF patients as compared to 1/98 (1%) in normal individuals. Patients with myelomonocytic skewing as compared to patients without skewing had higher white blood cell and blast cell counts, more frequent leukoerythroblastic features, but lower hemoglobin levels and platelet counts. The presence of myelomonocytic skewing was associated with a higher frequency of additional mutations, particularly in genes of the epigenetic and/or splicing machinery, and a significantly shorter survival (46 vs. 138 mo, *p* < 0.001). The results of this study show that the in vitro detection of myelomonocytic skewing can discriminate subgroups of patients with MF with a different phenotype, a different mutational profile and a different prognosis. Our findings may be important for the understanding and management of MF.

## 1. Introduction

Normal hematopoietic function is maintained by a well-controlled balance of myelomonocytic, megaerythroid and lymphoid progenitor cell populations. This balance may be skewed during pathologic conditions such as hematological malignancies, infections and autoimmunity [1,2,3,4,5,6,7]. Moreover, skewed hematopoiesis can be found in aged hematopoiesis [8]. Since semisolid in vitro cultures from peripheral blood mononuclear cells (PBMNC) of normal individuals usually contain a higher concentration of erythroid colonies (BFU-E), as compared to myelomonocytic colony-forming units (CFU-GM), this test may be useful for investigating skewed differentiation towards the myelomonocytic over erythroid commitment in patients [9]. In addition to genomic analyses, in vitro cultures may provide functional information and may help to more comprehensively characterize disturbed hematopoiesis in clonal hematologic disorders [9].

Patients with myelofibrosis (MF) have aberrant hematopoiesis due to molecular aberrations in a number of genes, including *JAK2*, *CALR* and *MPL* [10]. Moreover, analysis of the mutational landscapes of patients has shown that additional mutations can be found in a subset of patients and some of them, such as *ASXL1*, *SRSF2*, *EZH2*, *IDH1/2* or *U2AF1,* predicted an inferior outcome [11,12]. The role of myelomonocytic skewing in patients with myelofibrosis has not been studied so far. Our aim was to study the role of myelomonocytic skewing in patients with myelofibrosis.

## 2. Results

### 2.1. Progenitor Cells in Patients with MF

**Figure 1:**
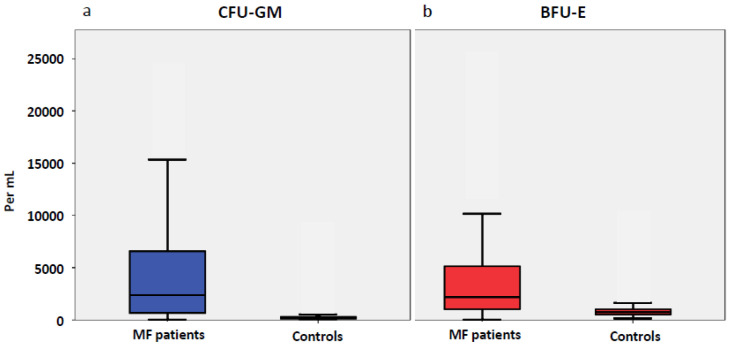
Box plots showing the distribution of circulating colony-forming units- granulocyte/macrophage (CFU-GM) (**a**) and burst-forming units-erythroid (BFU-E) (**b**) in patients with myelofibrosis and normal controls including median values, minimum values, maximum values, as well as upper and lower quartiles, respectively. Cultures were plated in duplicates at 100 × 10^3^ peripheral blood mononuclear cells (PBMNC)/mL. Aggregates with more than 40 translucent, dispersed cells were counted as CFU-GM. Bursts containing more than 100 red colored cells were scored as BFU-E. Progenitor cell data are expressed as mean values from duplicate cultures.

**Figure 2:**
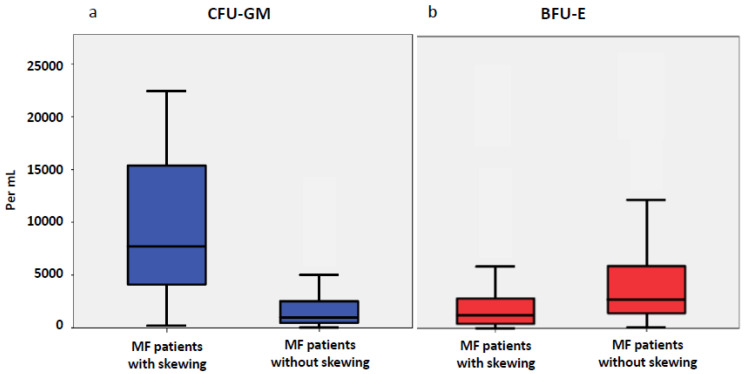
Box plots showing the distribution of circulating CFU-GM (**a**) and BFU-E (**b**) in patients with myelofibrosis with and without myelomonocytic skewing, including median values, minimum values, maximum values, as well as upper and lower quartiles, respectively.

In 81 patients with MF in vitro cultures were performed. The median numbers of myeloid and erythroid progenitor cells were significantly higher in the study patients than in the 98 healthy controls. The median CFU-GM was 1734/mL blood (range 47–115,467) and the median BFU-E was 2145/mL (11–34,776) (Figure 1, Table S1). The control group (n = 98) had a median CFU-GM of 202/mL blood (range 34–1413) and BFU-E of 735/mL (135–2779). The difference between the numbers in MF patients and in the control group was significant for both progenitor cell classes (CFU-GM, *p* < 0.001; BFU-E, *p* = 0.014). In the 81 MF patients studied there was a subgroup of patients in whom the number of CFU-GM exceeded the number of BFU-E. This myelomonocytic skewing as indicated by an inverse ratio of BFU-E/CFU-GM was found in 26/81 (32%) of patients with MF, whereas this phenomenon was rare in normal individuals (1/98, 1%). Figure 2a,b show the boxplots for circulating CFU-GM and BFU-E, respectively, in both subgroups. In patients with myelomonocytic skewing, the median CFU-GM was 8350/mL blood (range 1026–98,344) as compared to 910/mL (47–28,761) in patients without skewing (*p* < 0.001). The opposite changes were found in circulating erythroid progenitor cells, with a median number of 1234/mL (11–28,897) in patients with skewing, as compared to 2545/mL (94–34,776) in patients without skewing (*p* = 0.005).

### 2.2. Phenotype of MF Patients with and without Myelomonocytic Skewing

**Figure 3:**
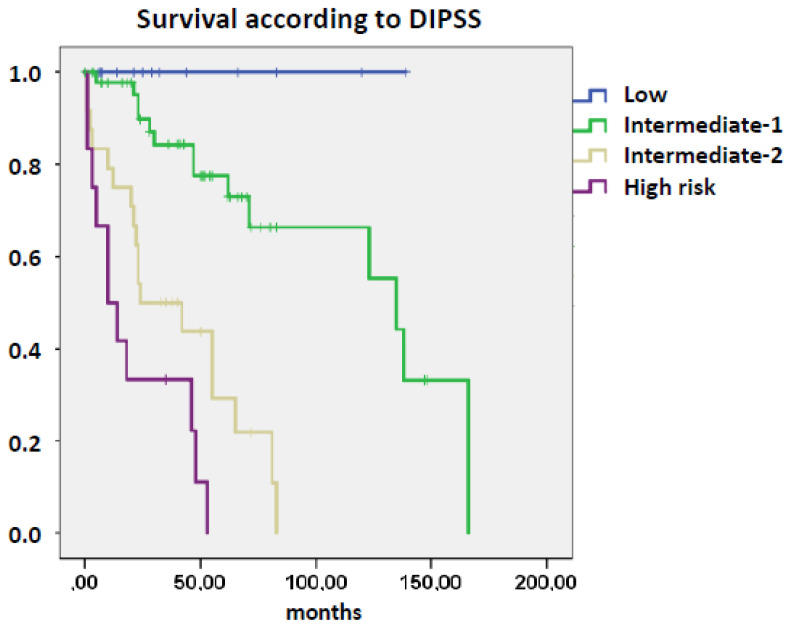
Overall survival in patients with myelofibrosis stratified by risk categories according to the Dynamic International Prognostic Scoring System (DIPSS) score.

As shown in Table 1, there were pronounced differences in the phenotype of patients with and without myelomonocytic skewing. Patients with myelomonocytic skewing had higher white blood cells (WBC) and blast cell counts in PB, more frequent leukoerythroblastic features but lower hemoglobin levels and platelet counts, respectively, as compared to patients without skewing. The absolute monocyte count (AMC) was not statistically different in both groups, and the proportion of patients with an AMC ≥ 10^9^/L was 11% (6/55) in patients without skewing as compared to 23% (6/26) in patients with skewing (*p* = 0.150). Figure 3 shows the survival curves of the 4 prognostic categories that were shown to predict survival in primary myelofibrosis by the International Working Group for Myeloproliferative Neoplasms Research and Treatment [13]. As one can see, the Dynamic International Prognostic Scoring System significantly discriminated four risk categories in our study, suggesting that the total patient cohort which was used in our study was representative regarding prognosis. When the two subgroups were analyzed separately, the proportion of patients within intermediate-2 and high-risk groups was significantly higher (58%) in patients with myelomonocytic skewing as compared to patients without an CFU-GM/BFU-E ratio ≥ 1 (16%).

### 2.3. Mutational Profile of MF Patients with and without Myelomonocytic Skewing

**Figure 4:**
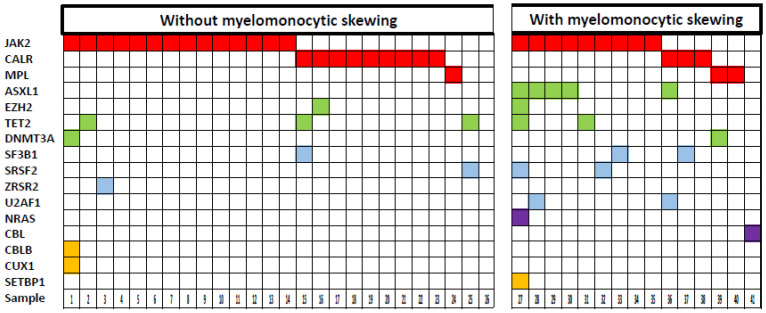
Comprehensive mutation status of genes in myelofibrosis patients with and without myelomonocytic skewing. Each column corresponds to one patient. Colored squares indicate mutated, white squares indicate wild-type genes. The colors of mutant genes indicate the most affected functional categories. Red, green, blue, purple and yellow represent the driver mutations, epigenetic regulators, spliceosome, RAS-pathway and other components, respectively.

Information on driver mutations determined by polymerase chain reaction (PCR) was available in 81 patients (Table S1). There was no significant difference between MF patients with and without myelomonocytic skewing, respectively, with regard to the distribution of MF driver mutations of *JAK2* (58% vs. 58%), *CALR* (19% vs. 27%), and *MPL* (8% vs. 2%), respectively (Table S1). Moreover, we could not find statistically significant differences in overall survival (OS) between different driver mutations in the whole cohort and in patients with or without skewing, respectively. However, as shown in Figure 4, the frequency of additional mutations determined by NGS was higher in patients with myelomonocytic skewing (11/15, 73%) as compared to patients without skewing (6/26, 23%; *p* = 0.002). In particular, genes of the splicing and/or epigenetic machinery were more frequently mutated in patients with as compared to patients without myelomonocytic skewing.

### 2.4. Survival of MF Patients with and without Myelomonocytic Skewing

**Figure 5:**
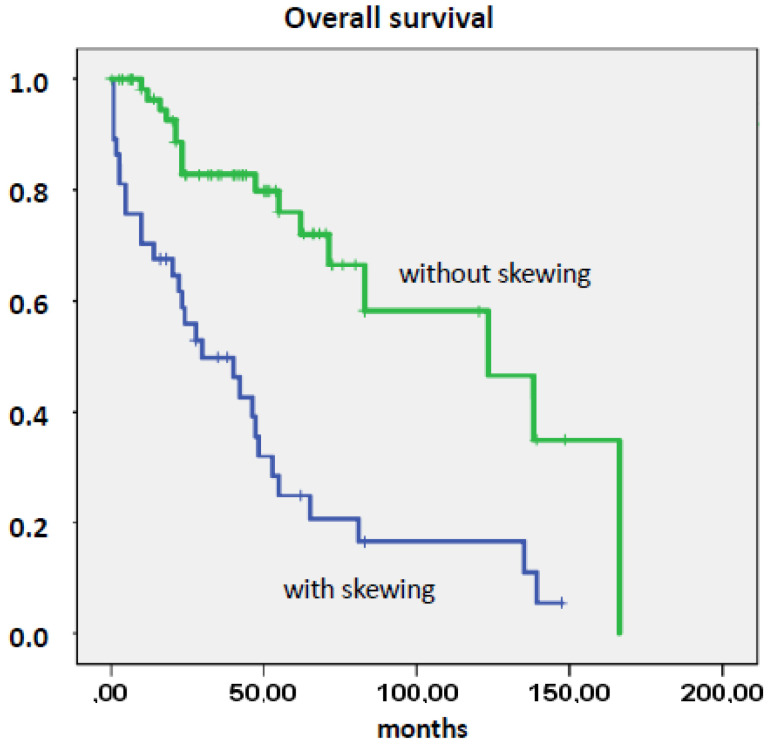
Overall survival of myelofibrosis patients stratified by the presence or absence of myelomonocytic skewing.

As shown in Figure 5, the presence of myelomonocytic skewing was associated with a significantly shorter survival. The median survival of patients with a CFU-GM/BFU-E ratio ≥1 was 46 months vs. 138 months in patients with a CFU-GM/BFU-E ratio <1 (*p* < 0.001). Table 2 shows the prognostic power of myelomonocytic skewing and established prognostic factors included in the DIPSS score. In a multivariate Cox regression analysis of overall survival, myelomonocytic skewing remained an independent prognostic factor (Table S2). Furthermore, an AMC ≥10^9^/L was a significant predictor of unfavorable outcome (Figure S1a), whereas the proportion of CD14 positive cells (>5%) in bone marrow (BM) had no prognostic impact (Figure S1b).

## 3. Discussion

It is well established in the literature that the number of hematopoietic progenitor cells is increased in peripheral blood of patients with myeloproliferative neoplasms (MPN). Levels of circulating hematopoietic progenitor cells are particular high in MF. In 1973, Paul Chervenick described the increased numbers of myeloid colony-forming cells in the peripheral blood of MF patients [14]. Subsequently, this finding was extended by a number of studies including ours, which demonstrated elevated numbers of circulating erythroid, megakaryocytic, and pluripotent progenitor cells in these patients [15,16,17,18]. The levels of circulating colony-forming cells were also significantly higher in MF than in normal controls in our study. Thus, the median CFU-GM levels were approximately eight times higher, and the median BFU-E approximately three times higher among MF patients than in controls. It is important to note that a subgroup of patients had rather low BFU-E numbers, but markedly increased CFU-GM levels resulting in a CFU-GM/BFU-E ratio of equal or more than one. Since the predominance of myelopoiesis over erythropoiesis may indicate some basic changes in the biology of this hematologic disorder, this observation prompted us to consider if patients with myelomonocytic skewing over the erythroid lineage were phenotypically, genotypically and prognostically different from patients without skewing.

The phenomenon of skewed myelopoiesis over erythropoiesis has been described in malignant and nonmalignant conditions in mice and man. Knockdown of *TET2* in cord blood CD34 (+) cells skews progenitor differentiation toward the granulomonocytic lineage, at the expense of lymphoid and erythroid lineages [2]. Deletion of *Tet2* in mice leads to an increased hematopoietic repopulating capacity with an altered differentiation, skewing towards monocytic/granulocytic lineages [1]. Other epigenetic regulators such as *ASXL1* have also been demonstrated to affect skewing of hematopoiesis. *Asxl1(−/−)* mice had a reduced hematopoietic stem cell (HSC) pool, and *Asxl1*(*−/−*) HSCs exhibited decreased hematopoietic repopulating capacity, with skewed cell differentiation favoring granulocytic lineage [3]. Furthermore, the splicing factors *SRSF2* and *U2AF1* seem to impact skewing. Mutations in both *SRSF2* and *U2AF1* cause abnormal differentiation by skewing granulo-monocytic differentiation towards monocytes [4]. On the other hand, nonmalignant conditions may also contribute to in vitro myelomonocytic skewing. It is well established that deregulated NF-κB activation contributes to the pathogenic processes of various inflammatory diseases. There is evidence from preclinical models that deregulation of NF-κB signaling promotes skewing of myelopoiesis over erythropoiesis. In mice, loss of IKKß skews differentiation towards myeloid over erythroid commitment and increases myeloid progenitor self-renewal and functional long-term hematopoietic stem cells. [6]. Moreover, Foxp3 deficiency in mice leads to exacerbated NF-kB activity and subsequent cytokine-mediated hyperproliferation of myeloid precursors [5].

The fact that the myeloid and erythroid progenitor cell compartment can be directly compared using in vitro culture of PBMNC makes this method particularly attractive to investigate this phenomenon. In our hands, we rarely find myelomonocytic skewing in PB from healthy individuals, but more frequently in patients with myeloid disorders. Particularly in myelodysplastic syndromes and chronic myelomonocytic leukemia, this phenomenon can be found in a high percentage of patients [19,20]. Both entities are disorders in which molecular aberrations of the epigenetic machinery, including *ASXL1* and *TET2*, seem to play a major pathophysiological role [21,22]. Interestingly, functional knockdown of *TET2* in CD34^+^/CD38^−^ caused a granulomonocytic expansion in vitro that was not observed in CD34^+^/CD38^+^ cells, suggesting that early dominance of the *TET2*-mutated clone in the immature CD34^+^/CD38^−^ compartment may participate in the granulomonocytic skewing that defines CMML [23]. Aging is characterized by clonal expansion of myeloid-biased hematopoietic stem cells, and recurrent somatic *TET2* mutations have been detected in normal elderly individuals with clonal hematopoiesis [24]. Additional mutations in MF including *ASXL1*, *SRSF2*, *EZH2*, *IDH1/2* and *U2AF1* have been shown to shorten the survival of patients with MF [11]. All these mutations were found in our MF patients with in vitro myelomonocytic skewing but were rare in patients without skewing; however, we are aware of the fact that NGS data were available only in a limited number of patients and that these data have to be confirmed in a larger cohort of MF patients. On the other hand, chronic inflammation seems to be an important trigger and driver of clonal evolution in MF [25]. One of the phenotypic differences between MF patients with and without skewing was the higher proportion of intermediate-2 and high-risk patients according to the DIPSS score in the group with a CFU-GM/BFU-E ≥1 [13]. It is intriguing to realize that the features of age, leukocytosis, anemia and constitutional symptoms which are included in DIPSS may be considered as either hematological consequences and/or promoting factors of myelomonocytic skewing over erythropoiesis.

Comparing the hematologic phenotype of our patients, we found that patients with myelomonocytic skewing had higher WBC and blast cell counts, but lower hemoglobin levels and platelet counts. The absolute monocyte count which may indicate myelomonocytic skewing in the blood was not statistically different in both groups, but the proportion of patients with an AMC ≥10^9^/L was twice (23% vs. 11%) in patients with skewing as compared to patients without skewing. The absolute monocyte count in MF patients has been shown to predict outcome [26,27]. Therefore, we also analyzed the prognostic role of the monocytic compartment in our patients. We could confirm that an AMC ≥10^9^/L in PB was associated with an inferior survival, however, the percentage of CD14 positive cells in BM did not predict an unfavorable outcome. This finding suggests that, similar to findings in CMML, aberrations of the monocytopoiesis may be more easily detected in PB as compared to BM.

The clinical relevance of our findings is clearly supported by our observation that myelomonocytic skewing was associated with an inferior outcome. Moreover, the prognostic impact of myelomonocytic skewing was independent of other established prognostic factors, since the effect retained significance in a multivariate Cox regression analysis. Considering the fact that additional mutations were significantly more frequent in MF patients with myelomonocytic skewing in this study, and the fact that additional mutations have been demonstrated to have an adverse impact on prognosis by others, one may speculate that myelomonocytic skewing detected by in vitro cultures may at a functional level reflect aberrations of hematopoiesis at the molecular level. Whatever the exact basis for myelomonocytic skewing may be, in vitro cultures may help to more comprehensively study hematopoiesis in patients with complex disturbances of blood formation.

## 4. Patients and Methods

### 4.1. Patients

This study is based on an Austrian clinicopathological registry, including patients diagnosed for MPN according to the 2008 WHO diagnostic criteria [28] between 1997 and 2020, which was created by clinicians and hematopathologists in the Departments of Hematology and Clinical Pathology at the Medical University of Vienna, Austria. The main eligibility criteria for entry into this study was the availability of representative, treatment-naïve BM biopsies (hematoxylin-eosin or Giemsa staining and silver impregnation after Gomori), confirming the diagnosis of myelofibrosis and the determination of circulating hematopoietic progenitor cells, which has been an integral part of the diagnostic work at our department in patients with suspected myeloid malignancies for many years, and which was in most cases performed at the time of diagnosis [9]. All the differential counts used for this study were manual differential counts. In total, 81 patients were included in this study, in 6/81 patients secondary MF was diagnosed. Diagnosis of secondary post PV/ET myelofibrosis required the demonstration of ≥2+ marrow fibrosis and/or clinical and morphological features according to International Working Group for Myeloproliferative Neoplasms Research and Treatment (IWG-MRT) criteria, including worsening of anemia, increase in splenomegaly either of newly palpable splenomegaly or more than 5 cm from baseline, overt leukoerythroblastosis or anisopoikilocytosis with tear-drop erythrocytes consistent with advanced PMF/myelofibrosis with myeloid metaplasia [29]. Leukoerythroblastosis was defined as the presence of immature cells of the myeloid series and nucleated red cells in the circulating blood. Samples were collected from MF patients after written informed consent according to the regulations of the ethics committee of the Medical University of Vienna (ethic code: 2115/2013). Clinical and laboratory routine parameters were obtained from patient records. This research has been approved by the ethic committee of the Medical University of Vienna on 20.04.2013 (ethic code: 2115/2013).

### 4.2. Colony Assay

The number of circulating CFU-GM and BFU-E, respectively, was assessed in semisolid cultures as previously described [30]. MNCs were isolated from PB of patients by Ficoll-Hypaque density gradient centrifugation (density 1.077 g/mL, 400 g for 40 min). The low-density cells were collected from the interface between density solution and plasma, washed twice, and resuspended in Iscove‘s modified Dulbecco’s medium (GIBCO, Paisley, Scotland). PBMNCs were cultured in 0.9% methylcellulose, 30% fetal calf serum (FCS; INLIFE, Wiener Neudorf, Austria), 10% bovine serum albumin (Behring, Marburg, Germany), α-thioglycerol (10^−4^ mol/L) and Iscove’s modified Dulbecco’s medium. For stimulation of progenitor cells, cultures were supplemented with recombinant human granulocyte-macrophage colony-stimulating factor (GM-CSF) (10 ng/mL; R&D Systems, Minneapolis, MN, USA), rh-interleukin-3 (10 U/mL; Novartis, Basel, Switzerland) and erythropoietin (EPO, 2 U/mL; Roche, Basel, Switzerland). Cultures were plated in duplicates at 100 × 10^3^ PBMNC/mL. Plates were incubated at 37 °C, 5% CO_2_, and full humidity. After a culture period of 14 days, cultures were examined under an inverted microscope. Aggregates with more than 40 translucent, dispersed cells were counted as CFU-GM. Bursts containing more than 100 red colored cells were scored as BFU-E. Progenitor cell data are expressed as mean values from cultures.

### 4.3. Molecular Analysis

Mutation analysis for MPN driver mutations included allele-specific polymerase chain reaction techniques to screen for Janus kinase 2 (*JAK2*), calreticulin exon 9 (*CALR*) and myeloproliferative leukemia virus oncogene (*MPL*) mutations [31]. Comprehensive mutational profiles in MF patients were determined by targeted re-sequencing as previously described [32]. DNA isolated from granulocytes or PBMCNs was processed using the TruSight Myeloid Panel kit (Illumina, San Diego, CA, USA) to generate indexed amplicon-based libraries. Equimolar amounts of libraries were pooled into multiplexes which were then sequenced 150bp paired-end on an Illumina HiSeq3000 instrument. Read alignment and variant calling was performed using the BaseSpace software (Illumina, San Diego, CA, USA). Variants called in transcribed regions or at splice sites were selected and further filtered for common variation. Other filters were adjusted for TruSight targeted sequencing and included insufficient sequencing read depth (<200) and low variant allele frequency (VAF < 0.05).

### 4.4. Statistical Analysis

The log-rank test was used to determine if individual parameters were associated with overall survival (OS). OS was defined as the time from sampling to death (uncensored) or last follow up (censored). A multivariate Cox regression analysis of overall survival was used to describe the relation between the event incidence, as expressed by the hazard function and a set of covariates. Dichotomous variables were compared between different groups with the use of the chi-square test. The Mann–Whitney U test was used to compare unmatched groups when continuous variables were not normally distributed. Results were considered significant at *p* < 0.05. Statistical analyses were performed with the SPSS version 19.0.0 (SPSS Inc., Chicago, IL, USA); the reported *p* values were 2-sided.

## 5. Conclusions

In conclusion, we report for the first time a study which investigates the phenomenon of myelomonocytic skewing as determined by semisolid in vitro cultures in patients with MF. We can show that the presence or absence of myelomonocytic skewing can discriminate these patients regarding clinical, phenotypic and molecular characteristics. Moreover, the clinical relevance of our findings is further supported by the different outcome of both groups, stratified by whether or not patients showed myelomonocytic skewing. More generally, we think that myelomonocytic skewing as determined by semisolid in vitro cultures may still be an important functional method to complement molecular analyses and to comprehensively study disturbed hematopoiesis in various conditions.

## Figures and Tables

**Table 1:** Phenotypic characteristics of 81 patients with myelofibrosis stratified by whether or not they had myelomonocytic skewing in semisolid cultures.

Variables	All MF Patients (*n* = 81)	MF Patients with Myelomonocytic Skewing (*n* = 26)	MF Patients without Myelomonocytic Skewing (*n* = 55)	*p*-Value
Age in years; median (range)	66.0 (25–88)	69 (42–86)	64 (25–88)	0.116
Sex (Male); n (%)	45/81 (56%)	18/26 (69%)	27/55 (49%)	0.089
WBC × 10⁹/L; median (range)	8.4 (2.4–96)	12.1 (3.1–96)	8.2 (2.4–44.7)	0.036
Hemoglobin g/dL, median (range)	11.9 (6.6–16.3)	9.6 (6.6–14.3)	13.0 (8.3–16.3)	<0.001
Platelets × 10⁹/L; median (range)	424 (15–1584)	244 (15–1450)	516 (18–1584)	0.002
PB blasts %; median (range)	0 (0–10)	0.5 (0–10)	0 (0–2)	0.018
Leukoerythroblastic change (%)	22/81 (27%)	11/26 (42%)	11/55 (20%)	0.035
Monocytes × 10⁹/L; median (range)	0.50 (0–16.3)	0.51 (0–16.3)	0.50 (0–1.6)	0.704
Intermediate-2 and high-risk category according to DIPSS (%)	24/81 (30%)	15/26 (58%)	9/55 (16%)	<0.001
Post PV/ET MF (%)	6/81 (7%)	2/26 (8%)	4/55 (7%)	0.946

MF: myelofibrosis; WBC: white blood cell count; PB: peripheral blood; DIPPS: Dynamic International Prognostic Scoring System.

**Table 2:** Univariate analysis of single prognostic parameters in patients with myelofibrosis.

Factors	Factor Present Md OS (mo)	Factor Absent Md OS (mo)	Hazard Ratio	*p*-Value (Log-Rank)
Skewing present	46	138	3.98	<0.001
WBC > 25 × 10^9^/L	5	83	3.66	0.025
Hb < 10 g/dL	46	139	5.39	<0.001
PLT < 100 × 10^9^/L	21	83	2.95	0.007
PB Blasts present	47	123	1.95	0.103
Age > 65 years	53	139	3.66	0.001

OS, overall survival; WBC, white blood cell count; Hb, hemoglobin; PLT, platelet count, PB, peripheral blood. The log-rank test was used to determine if individual parameters were associated with OS.

## Supplementary Materials

The following are available online at https://www.mdpi.com/2072-6694/12/8/2291/s1, Table S1: Circulating CFU-GM and BFU-E numbers, CFU-GM/BFU-E ratio and driver mutation status in patients with myelofibrosis, Table S2: Multivariate Cox regression analysis of overall survival, Figure S1: Overall survival of myelofibrosis patients stratified by the presence or absence of absolute monocytosis ≥ 10^9^/L (**a**) and the proportion of CD14 positive bone marrow cells > 5% (**b**).
