# Supplementary material for: Myelomonocytic Skewing In Vitro Discriminates Subgroups of Patients with Myelofibrosis with A Different Phenotype, A Different Mutational Profile and Different Prognosis
Source: Cancers (Basel). 2020 Aug 14;12(8):2291. doi: 10.3390/cancers12082291 (PMC7464756; doi:10.3390/cancers12082291)
Supplement: Supplementary file 1 [file cancers-12-02291-s001.pdf]

cancers

MDPI

Article

Myelomonocytic Skewing in Vitro Discriminates Subgroups of Patients with Myelofibrosis with A Different Phenotype, A Different Mutational Profile and Different Prognosis

Klaus Geissler [1,2,*], Bettina Gisslinger [3], Eva Jäger [4], Roland Jäger [5], Ana Iris Schiefer [6], Edith Bogner [5], Elisabeth Fuchs [5], Fiorella Schischlik [5], Donat Alpar [5], Ingrid-Simonitsch-Klupp [6], Robert Kralovics [4,5] and Heinz Gisslinger [3]

1 Medical School, Sigmund Freud University, 1020 Vienna, Austria
2 Department of Internal Medicine V with Hematology, Oncology and Palliative Care, Hospital Hietzing, 1130 Vienna, Austria
3 Division of Hematology and Hemostaseology, Department of Internal Medicine I, Medical University of Vienna, 1090 Vienna, Austria; bettina.gisslinger@meduniwien.ac.at (B.G.); heinz.gisslinger@meduniwien.ac.at (H.G.)
4 Department of Laboratory Medicine, Medical University of Vienna, 1090 Vienna, Austria; eva.jaeger@akhwien.at (E.J.); rkralovics@cemm.oeaw.ac.at (R.K.)
5 CeMM Research Center for Molecular Medicine of the Austrian Academy of Sciences, 1090 Vienna, Austria; roland.jaeger@meduniwien.ac.at (R.J.); edith_bogner@hotmail.com (E.B.); elisabeth.fuchs@edumail.at (E.F.); fiorella.schischlik@nih.gov (F.S.); dalpar@cemm.oeaw.ac.at (D.A.)
6 Clinical Institute of Pathology, Medical University of Vienna, 1090 Vienna, Austria; ana-iris.schiefer@meduniwien.ac.at (A.I.S.); ingrid.simonitsch-klupp@meduniwien.ac.at (I.-S.-K.)
* Correspondence: klaus.geissler@wienkav; Tel.: +43-01-80110-3122; Fax: +43-01-80110-2671

Supplementary

Table S1. Circulating CFU-GM and BFU-E numbers, CFU-GM/BFU-E ratio and driver mutation status in patients with myelofibrosis.

Pat Nr	Sex	Age	Driver Mutation Status	Follow up Time in Months	CFU-GM/ml	BFU-E/ml	CFU-GM/BFU-E Ratio
1	m	71	*JAK2* wt	12	222	24737	0.01
2	m	73	*JAK2* mut	7	81	1013	0.08
3	m	48	*JAK2* mut	43	134	1562	0.09
4	f	74	*JAK2* mut	23	73	640	0.11
5	f	50	*JAK2* mut	33	473	4476	0.11
6	m	60	*JAK2* mut	63	1533	12209	0.13
7	m	75	*JAK2* mut	21	196	1500	0.13
8	m	67	*JAK2* mut	40	332	2363	0.14
9	f	50	*JAK2* mut	7	301	2040	0.15
10	m	72	*JAK2* mut	20	192	1267	0.15
11	f	44	*JAK2* mut	32	586	3320	0.18
12	m	71	*JAK2* mut	52	836	4251	0.2
13	f	68	*JAK2* mut	71	320	1469	0.22
14	f	45	*JAK2* mut	29	1114	4129	0.27
15	m	69	*CALR* mut	10	1364	5072	0.27
16	f	58	Triple neg	44	373	1325	0.28
17	f	72	*JAK2* wt	23	6938	24877	0.28
18	f	45	*JAK2* mut	14	448	1627	0.28
19	m	52	*CALR* mut	6	1152	4018	0.29
20	f	82	*CALR* mut	76	563	1809	0.31
21	f	88	*JAK2* mut	50	420	1370	0.31
22	f	58	*JAK2* mut	51	1803	5900	0.31
23	f	53	*JAK2* mut	21	513	1665	0.31

24	m	46	*JAK2* mut	120	538	1746	0.31
25	f	35	*CALR* mut	41	1663	5088	0.33
26	m	25	*CALR* mut	43	2822	8248	0.34
27	m	67	Triple neg	0	154	450	0.34
28	f	64	*JAK2* mut	7	989	2748	0.36
29	m	49	*CALR* mut	62	3082	8386	0.37
30	f	54	*JAK2* mut	72	2022	5184	0.39
31	f	75	*CALR* mut	3	1734	4167	0.42
32	f	78	*JAK2* mut	166	747	1737	0.43
33	f	45	*JAK2* wt	55	910	1918	0.47
34	f	64	*JAK2* mut	66	478	1014	0.47
35	m	46	*JAK2* wt	24	2876	5941	0.48
36	m	36	*JAK2* mut	83	47	94	0.5
37	m	69	*JAK2* mut	40	2251	4480	0.50
38	m	82	*CALR* mut	36	3349	6658	0.50
39	m	70	*JAK2* mut	54	3214	6022	0.53
40	f	66	*MPL* mut	83	1767	3304	0.53
41	f	41	*CALR* mut	66	1395	2545	0.55
42	f	76	*JAK2* mut	51	784	1395	0.56
43	m	61	*CALR* mut	50	2449	4194	0.58
44	f	66	*CALR* mut	138	1239	2145	0.58
45	m	55	*CALR* mut	7	4045	6849	0.59
46	m	55	*JAK2* mut	139	875	1416	0.62
47	m	63	*CALR* mut	72	701	1069	0.66
48	f	82	*JAK2* mut	80	2570	3674	0.70
49	m	51	*JAK2* mut	148	6349	8997	0.71
50	m	73	*JAK2* mut	68	1103	1538	0.72
51	f	78	*CALR* mut	47	576	797	0.72
52	m	70	*JAK2* mut	35	1682	2181	0.77
53	f	52	*CALR* mut	4	785	1009	0.78
54	f	86	Triple neg	10	28761	34776	0.82
55	m	57	*JAK2* mut	123	6754	7085	0.95
56	m	62	*JAK2* mut	30	10328	8977	1.15
57	f	42	*CALR* mut	28	1681	1225	1.37
58	f	75	*JAK2* mut	83	4034	2800	1.44
59	f	74	*JAK2* mut	53	4840	2728	1.77
60	m	58	*JAK2* mut	81	9375	5239	1.79
61	f	68	*JAK2* mut	46	3723	1887	1.97
62	f	71	*JAK2* mut	23	4096	1946	2.1
63	m	73	*JAK2* mut	5	61923	28897	2.14
64	m	72	*JAK2* wt	14	5500	2200	2.5
65	f	53	*JAK2* mut	62	1706	552	3.09
66	m	52	*CALR* mut	147	10639	3224	3.30
67	m	60	*MPL* mut	139	3270	688	4.75
68	f	68	*CALR* mut	40	13231	2623	5.04
69	f	71	*JAK2* mut	18	4770	875	5.45
70	m	55	*JAK2* mut	16	1026	134	7.66
71	m	86	*CALR* mut	10	22418	2842	7.89
72	m	75	*JAK2* mut	1	12242	1243	9.85
73	m	71	*JAK2* mut	65	98344	5887	16.70
74	m	61	*MPL* mut	55	17758	539	32.94
75	m	78	*JAK2* mut	35	7700	172	44.76
76	m	70	*JAK2* wt	2	53033	851	62.32
77	m	59	*JAK2* mut	38	4000	57	70.18
78	m	58	*CALR* mut	3	16524	229	72.16
79	m	81	*JAK2* wt	20	9000	95	94.73
80	m	58	Triple neg	47	11260	97	116.08
81	m	81	*JAK2* mut	22	2456	11	223.27

Mut, mutated; wt, wildtype; NA, not available; CFU-GM, colony-forming unit granulocyte/macrophage; BFU-E, burst-forming unit erythroid.

Table S2. Multivariate Cox regression analysis of overall survival.

Parameter	Hazard Ratio	95% Confidence Interval	P-Value
Skewing present	3.31	1.22-8.96	0.019
WBC >25 x 10^9/L	4.50	0.93-21.82	0.062
Hb <10 g/dL	2.75	0.92-8.27	0.071
PLT <100 x 10^9/L	3.39	1.24-9.30	0.018
PB Blasts present	1.55	0.6-3.97	0.363
Age >65 years	3.98	1.96-10.85	0.007

WBC, white blood cell count; Hb, hemoglobin; PLT, platelet count, PB, peripheral blood; The proportional hazards model was used to evaluate the prognostic importance of each factor while simultaneously considering the effects of other covariates. For the purpose of clinical utility, continuous covariates were regarded as dichotomous, with categories determined based on consideration of previously reported cut off points in this disease. Covariates including skewing, WBC, Hb, PLT, PB blasts and age, respectively, have been used to make the adjustments.

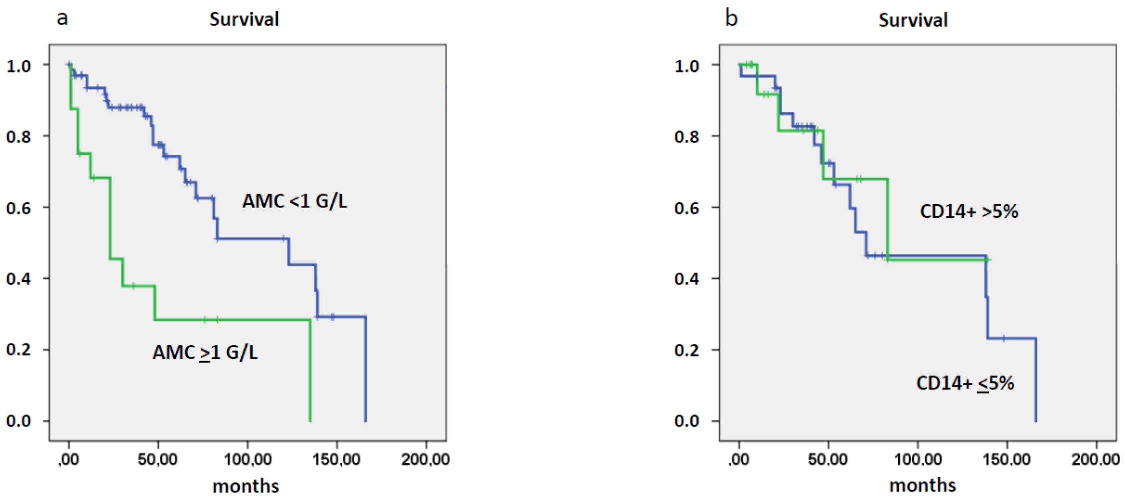

Figure S1. Overall survival of myelofibrosis patients stratified by the presence or absence of absolute monocytosis ≥ 1 G/L (**a**) and the proportion of CD14 positive bone marrow cells > 5% (**b**). AMC, absolute monocyte count; Figure S1a: median overall survival in patients with AMC ≥ 1 G/L 23 months vs. 83 months in patients with AMC <1 G/L ($p = 0.006$). Figure S1b: median overall survival in patients with CD14 pos cells > 5% 83 months vs. 65 months in patients with CD14 pos cells ≤ 5% ($p = 0.566$).
